# Quantitative analysis of aquaporin-4 (AQP4) and myelin oligodendrocyte glycoprotein (MOG) antibodies titres: correlation with relapses

**DOI:** 10.1093/braincomms/fcaf312

**Published:** 2025-08-22

**Authors:** Martina Nasello, Mark Woodhall, Huiru Xue, Victor Mgbachi, Ruth Geraldes, Maria Isabel Leite, Patrick Waters, Jacqueline Palace

**Affiliations:** Nuffield Department of Clinical Neuroscience, University of Oxford, Oxford, UK; Department of Neurosciences, Mental Health and Sensory Organs, Centre for Experimental Neurological Therapies (CENTERS), Sapienza University of Rome, Rome, Italy; Nuffield Department of Clinical Neuroscience, University of Oxford, Oxford, UK; Nuffield Department of Clinical Neuroscience, University of Oxford, Oxford, UK; Department of Neurology, First Hospital of Shanxi Medical University, Taiyuan, China; Nuffield Department of Clinical Neuroscience, University of Oxford, Oxford, UK; Nuffield Department of Clinical Neuroscience, University of Oxford, Oxford, UK; Nuffield Department of Clinical Neuroscience, University of Oxford, Oxford, UK; Nuffield Department of Clinical Neuroscience, University of Oxford, Oxford, UK; Nuffield Department of Clinical Neuroscience, University of Oxford, Oxford, UK

**Keywords:** neuromyelitis optica spectrum disorder, MOGAD, immunoglobulins, antibody titres

## Abstract

Detection of antibodies against aquaporin-4 (AQP4-IgG) and myelin oligodendrocyte glycoprotein (MOG-IgG) is essential for diagnosis of AQP4-IgG+ neuromyelitis optica spectrum disorder and myelin oligodendrocyte glycoprotein antibody-associated disease. Uncertainties persist regarding the clinical significance of serum antibody titres to predict relapses. We aimed to analyse the trend of serum AQP4-IgG and myelin oligodendrocyte glycoprotein antibody-IgG titres during relapses and periods of clinical stability. In this retrospective study we analysed serum AQP4-IgG and myelin oligodendrocyte glycoprotein antibody -IgG titres from live cell-based assays from the Oxford Autoimmune Neurology Diagnostic Laboratory for the UK specialist NMO Service between February 2010 and June 2024. We recruited seropositive AQP4-neuromyelitis optica spectrum disorder and myelin oligodendrocyte glycoprotein antibody-associated disease patients with serum samples available within 30 days of an attack (‘attack’) and at least one another sample for comparison within 1 year (‘pre-attack’ and ‘post-attack’). Up to 3 further serum samples were selected within 2, 3 or 4 years after the attack. 117 attacks in 92 AQP4-IgG+ patients (81.5% female) and 127 attacks in 111 myelin oligodendrocyte glycoprotein antibody-IgG+ patients (62.2% female) had appropriate samples. Antibody titres significantly increased from ‘pre-attack’ to ‘attack’, decreased from ‘attack’ to ‘post-attack’, and remained stable from 2 to 4 years after attacks. Long-term immunosuppressant treatments induced a further decrease in titres during remission in both cohorts. In 40% of samples in both groups there was an increase in titre at relapse. A ≥ 2-fold increase in AQP4-IgG titres had an Odds Ratio (OR) of 6.59% and 91.5% specificity of being associated with a relapse, in myelin oligodendrocyte glycoprotein antibody-IgG+ an OR of 4.87% and 88.6% specificity. 29% (26/92) AQP4-IgG+ patients became ‘seronegative’ during follow: most patients (64%) had low attack titres (≤100), and the time to seroreversion related the attack titre. In contrast, 41% (46/111) myelin oligodendrocyte glycoprotein antibody-IgG+ patients became ‘seronegative’, mostly within the first year after the attack regardless of attack titre. In conclusion, in 60% of longitudinal serum samples from patients with AQP4-IgG or myelin oligodendrocyte glycoprotein antibody-IgG there is no increase in antibody titre. When there is an increase, it is most often at relapse. A ≥ 2-fold increase in titres should be a risk particularly in case of treatment de-escalation, non-adherence to treatment or if a pseudo-relapse is suspected.

## Teaser

Nasello *et al.* report titres of antibodies against acquaporin-4 (AQP4-IgG) and myelin oligodendrocyte glycoprotein (MOG-IgG) increase in 40% of Neuromyelitis Optica Spectrum Disorder and MOG-IgG-associated disease relapses, remaining stable 2–4 years post-attacks. A ≥ 2-fold titres increase should be a risk particularly in case of non-adherence to treatment or suspected pseudo-relapse.

## Graphical Abstract

**Graphical Abstract:**
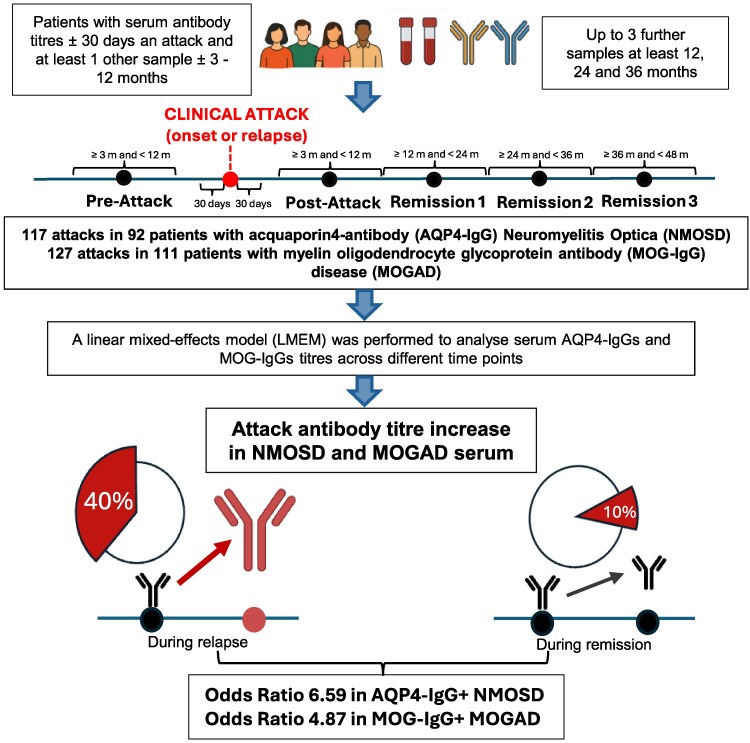

## Introduction

Neuromyelitis Optica Spectrum Disorders (NMOSD) and Myelin oligodendrocyte glycoprotein (MOG) antibody-associated disease (MOGAD) are autoimmune disorders affecting the central nervous system.^1,2^ The detection of specific antibodies, respectively against the water channel acquaporin-4 (AQP4) and MOG, is critical for diagnosis, but the role of anti-AQP4 and -MOG antibody titres in managing NMOSD and MOGAD remains unclear.^3,4^

In these conditions, disability is strictly related to an incomplete or partial recovery from clinical attacks,^5^ making the identification of reliable biomarkers to predict or confirm relapses essential to improve prognosis and guide treatment choices.

Although immunoglobulins G antibodies against AQP4 (AQP4-IgGs) appear to play a significant role in the development of inflammatory/demyelinating lesions,^6^ the correlation of serum titres within an individual with clinical attacks and utility in predicting disease activity remain uncertain.^7^ To date, monitoring serum AQP4-IgG levels as an identifier and predictor of relapses has shown inconclusive results, with varying results in different studies.^4,8^

Persistence of immunoglobulins G antibodies against MOG (MOG-IgGs) is associated with an increased risk of relapsing disease course in MOGAD.^3,9^ However, the use of serum titres in predicting relapses and confirming an attack also remains uncertain.^10^

Therefore, we analysed the trend of serum AQP4-IgGs and MOG-IgGs titres over time, specifically to quantify titres during relapses and during periods of clinical stability, and to assess the potential use of these antibody titres to predict or confirm an attack.

## Materials and methods

### Identification of samples

We retrospectively analysed serum AQP4-IgGs and MOG-IgGs titres by live cell-based assay (CBA) in the Oxford Autoimmune Neurology Diagnostic Laboratory on patients followed in the national Oxford NMO Service. Sera were screened at a dilution of 1:20 for AQP4-IgG or 1:200 for MOG-IgG. If positive they were serially diluted in doubling dilutions. The highest dilution to give a positive score is considered the titre. Titres ≥ 1:20 for AQP4-IgG or titres ≥ 1:200 for MOG-IgGs were reported as positive. Patients were included if they had a diagnosis of AQP4-IgG-NMOSD or MOGAD, according to the most recent diagnostic criteria.^1,2^ To assess the association of antibody titres with clinical attacks, we only included patients who had serum samples available within 30 days before or after an attack (symptoms onset) and at least one other sample for comparison collected between 3 and 12 months from an attack (before or after). We set the time-frame for the second sample at least 3 months after the attack to reduce the effect of acute treatment on titres. In these patients, we included up to three further samples to assess the antibody titre trend during clinical stability. The samples were at least 12, 24 or 36 months from the clinical attack without relapse in between. In 22 AQP4-IgG+ patients, several serum samples were available in the same time window, in these cases the modal antibody titre was selected.

**Figure 1:**
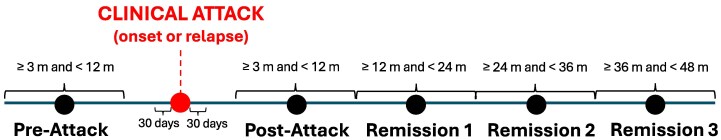
**Timeline of all time points.** Timeline of all time points: pre-attack, clinical attack, post-attack, remission 1, remission 2 and remission 3. Above parenthesis: time of serum collection from clinical attack. Abbreviation: m, months.

This categorization provided 6 time points: ‘pre-attack’ (≥ 3 months and <12 months before a relapse), ‘attack’ (within 30 days before/after a clinical attack), ‘post-attack’ (≥ 3 months and <12 months after a clinical attack), ‘remission 1’ (≥12 months and <24 months after a clinical attack), ‘remission 2’ (≥ 24months and <36 months after clinical attack) and ‘remission 3’ (≥36 months and <48 months after a clinical attack) (Fig. 1).

A clinical attack was defined as new onset of neurological symptoms and/or exacerbation of pre-existing ones, radiologically confirmed by new/enlarging or enhancing T2 hyperintense lesions on MRI compared to baseline imaging,^11,12^ or by worsening in visual acuity or on Optical Coherence Tomography for optic neuritis (ON) (corresponding to optic disc swelling acutely or an inter-eye difference in the macular ganglion cell inner plexiform layer, mGCIPL, of >4% or >4 μm, or in the peripapillary retinal nerve fibre layer, pRNFL, of >5% or >5 μm, within 3 months after onset).^13^ Where ON attacks were borderline and no MRI was available objective change in pupil responses, reliable visual field defects and subsequent atrophy on OCT in the chronic (≥3 months) phase of ON were used to confirm an attack.^13^ Each clinical attack was extracted from our database, where relapses were collected prospectively.

Information on each clinical attack and the relevant immunosuppressive therapies were collected to evaluate their impact on antibody titres. These included clinical phenotype, severity of clinical attack at nadir (according to the expanded disability status scale, EDSS), the number of a specific clinical attack (i.e. onset attack or attack 2, 3 or 4 for example), and use of immunosuppressant treatment (IST) after/during the attack. Clinical phenotypes of attacks were classified as ON, transverse myelitis (TM), brainstem syndrome (BS), acute disseminated encephalomyelitis (ADEM) or cerebral attacks (CA). When two or more of the phenotypes were present in an individual attack it was labelled as multifocal (MF). Long-term ISTs were classified as traditional IST (oral steroids lasting longer than 12 months, azathioprine, mycophenolate, methotrexate, and IVIg) or B cell depleting treatments (rituximab and ofatumumab). For MOG-IgG+ patients only an additional IST category was included: a short course of oral steroids lasting less than 12 months and more than 2 months. Sex, age at onset, age at attack, time since last attack and disease course (monophasic or relapsing) were also collected.

### Ethical committee

Participating patients signed a written informed consent to collect their clinical data according to the Oxford Research Ethics (South Central–Oxford C Research Ethics Committee code 21/SC/0353).

### Statistical analysis

All analyses of AQP4-IgG and MOG-IgG titres were performed on a logarithmic base-2 scale because of the doubling dilution steps for the titration assay. A linear mixed-effects model (LMEM) with random intercepts and random slopes was performed to analyse serum AQP4-IgG and MOG-IgG titres across different time points. Pairwise comparisons were conducted between the following phases: ‘pre-attack’ versus ‘attack’, ‘attack’ versus ‘post-attack’, ‘remission 1’ versus ‘remission 2’ and ‘remission 2’ versus ‘remission 3’. We identified different subgroups based on the IST used (short course of oral steroids, traditional ISTs including corticosteroids, or B cell depleting treatments), and in these subgroups a multilevel comparison was also performed to compare the phases: ‘attack’, ‘post-attack’, ‘remission 1’, ‘remission 2’ and ‘remission 3’. The dependent variable was serum antibody titre.

Interactions with disease course, number of clinical attacks, attack EDSS, age at onset, age at attack, time since last attack and IST as independent variables were incorporated to investigate whether the influence of time points on antibody titres varied across these categories. For those comparisons including ‘attack’ samples, having the serum sample collected before 30 days of symptoms onset have been incorporated as independent variable to see any difference of time collection on titres change.

*Post hoc* pairwise comparisons were conducted using the ‘emmeans’ package in R Studio Statistical Software (version 1.10.4).

All statistical analyses were performed using R Studio Statistical Software (version 2024.9.1.394; Posit Software, Boston, USA).

## Results

### Cohort description

**Figure 2:**
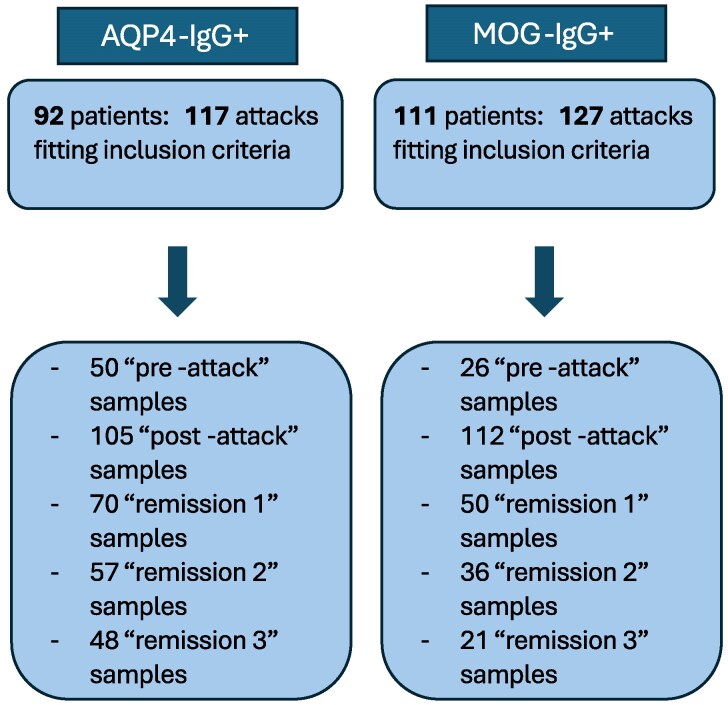
**Enrolment profile**. Description of the enrolment profile of both cohorts, AQP4-IgG+ and MOG-IgG+ . Above: total number of patients and attacks included. Below: total number of serum samples included for each time point.

We screened 287 AQP4-IgG+ and 369 MOG-IgG+ patients for a total of 916 AQP4-IgG+ and 877 MOG-IgG+ attacks. We included 92 AQP4-IgG+ patients (81.5% female, median age at onset 43 years, inter-quartile range, IQR 28–56, median time of follow-up 6 years, IQR 3–8), who had a serum sample taken within 30 days of 117 attacks and had a second sample taken within 3–12 months of the attack. Up to three further samples from each individual that fell into the pre-selected time-frames were included in the study. In addition to the 117 attack samples, another 330 samples were included: 50 ‘pre-attack’ samples, 105 ‘post-attack’ samples, 70 ‘remission 1’ samples, 57 ‘remission 2’ samples, 48 ‘remission 3’ samples. Similarly, in 111 MOG-IgG+ individuals (62.2% female, median age at onset 34 years, IQR 24–42, median time of follow-up 3 years, IQR 1–4) 127 attack samples and 245 other samples were identified: 26 ‘pre-attack’ samples, 112 ‘post-attack’ samples, 50 ‘remission 1’ samples, 36 ‘remission 2’ samples, and 21 ‘remission 3’ samples (Fig. 2). Baseline features are shown in Table 1: 33.7% AQP4-IgG+ patients and 62.2% MOG-IgG+ patients had no relapses at follow-up (median time 4 years, IQR 2–6 years). Most clinical attacks included were relapses for AQP4-IgG+ (71%) and onset attacks for MOG-IgG+ (60%), TM was the most representative phenotype for AQP4-IgG+ (47%) and ON for MOG-IgG+ (63%). Clinical attacks included had a median EDSS at nadir of 4 (IQR 3–6.5) for AQP4-IgG+ and 3 (IQR 2–4) for MOG-IgG+ . The majority of AQP4-IgG+ cohort was treated with traditional ISTs (azathioprine and mycophenolate, 68%). Most of MOG-IgG+ cohort (58%) were treated with oral cortico-steroids. Only 26% received a short course <12 months (median time 7 months, IQR 4–9 months). 20 (18%) MOG-IgG+ and 1 (1%) AQP4-IgG+ patients did not receive long-term IST. Characteristics of serum samples (time-from attack and titre) are described in Supplementary Table 1. Serum sample pairs from different stages of disease course in patients with either AQP4-IgG+ NMOSD or MOGAD were evaluated for a change in titre. In 144 serum samples, from 67 AQP4-IgG+ patients, there was an increase of at least 2-fold in 27 (18.7%) samples, a decrease in 27 (18.7%) samples and 90 (62.5%) remained stable. Similarly in 70 samples from 48 patients with MOGAD, 15 (21.4%) increased, 12 (17.1%) decreased and 43 (61.4%) remained stable.

**Table 1:** Demographic and baseline clinical characteristics of patients

Characteristics	AQP4-IgG+ Patients (*N* = 92)	MOG-IgG+ Patients (*N* = 111)
Sex (%)	Female (81.5%)	Female (62.2%)
Age at onset, median (IQR)	43 years (28–56)	34 years (24–42)
Disease course (%)	Relapsing (66.3%)	Relapsing (37.8%)
Clinical attacks included, N (%)	117	127
Onset attacks	34 (29%)	76 (60%)
Relapses (second—fifth)	55 (47%)	44 (35%)
Relapses (>sixth)	28 (24%)	7 (5%)
EDSS clinical attacks, median (IQR)	4 (3–6.5)	3 (2–4)
Phenotypes clinical attacks, *N* (%)		
TM	55 (47%)	18 (14%)
ON	27 (23%)	80 (63%)
BS	10 (9%)	2 (2%)
ADEM	0 (0%)	5 (4%)
CA	3 (3%)	1 (1%)
MF	22 (19%)	21 (17%)
Long-term IST after clinical attacks, *N* (%)		
Azathioprine	43 (38%)	9 (8%)
Mycophenolate	34 (30%)	17 (15%)
Methotrexate	4 (4%)	0 (0%)
IVIg	0 (0%)	1 (1%)
B cell depleting	26 (23%)	0 (0%)
Oral steroids ≥12 m	6 (5%)	37 (32%)
Oral steroids <12 m	0 (0%)	30 (26%)
No treatment	1 (1%)	20 (18%)

AQP4, acquaporin-4; MOG, myelin oligodendrocyte glycoprotein; IgG, immunoglobulin G; *N*, numbers; IQR, inter-quartile range; EDSS, expanded disability status scale; TM, transverse myelitis; ON, optic neuritis; BS, brainstem syndrome; ADEM, acute demyelinating encephalomyelitis; CA, cerebral attacks; MF, multifocal attacks; IST, immunosuppressant treatment; IVIg, intravenous immunoglobulin; m, months.

### Antibody titres increase in serum from ‘pre-attack’ to ‘attack’

**Figure 3:**
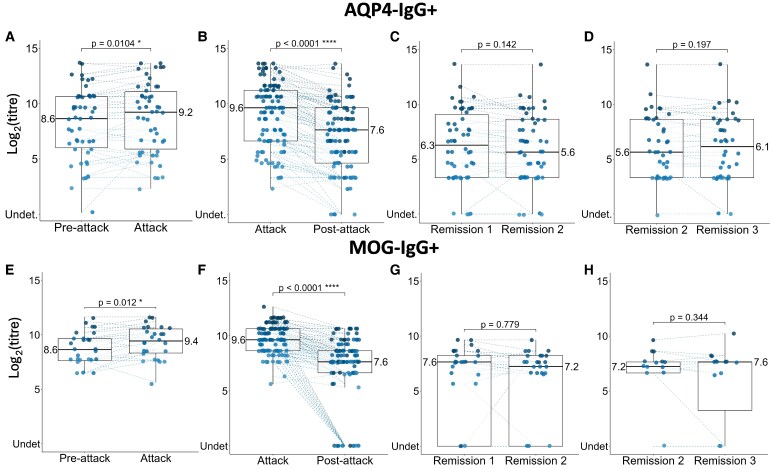
**Box plots of the distribution and variability of titres across the different time points**. Box plots of the distribution and variability of titres across the different time points for AQP4-IgG+ (A–D) and MOG-IgG+ (E–H), during attacks (A, B, E and F) and clinical stability (C, D, G and H). Each box plot show statistical significance of antibody titres change by LMEM: ‘Pre-Attack’ versus ‘Attack’ (*N* = 50 relapses for AQP4-IgG+, A, *N* = 26 relapses for MOG-IgG+, E), ‘Attack’ versus ‘Post-Attack’ (*N* = 105 attacks for AQP4-IgG+, B, *N* = 112 attacks for MOG-IgG+, F), ‘Remission 1’ versus ‘Remission 2’ (*N* = 54 sequences for AQP4-IgG+, C, *N* = 29 sequences for MOG-IgG+, G), and ‘Remission 2’ versus ‘Remission 3’ (*N* = 40 sequences for AQP4-IgG+, D, *N* = 15 sequences for MOG-IgG+). Each point represents the titre value (in log2 scale) of a single attack, coloured differently for each value. Dashed lines combine the antibody titres for the same attack. Next to the label of each box plot is the median value of the antibody titres for that time point. Abbreviations: Undet, undetectable; LMEM, linear mixed effect model. * = *P* ≤ 0.05, ** = *P* ≤ 0.01, *** = *P* ≤ 0.001, **** = *P* ≤ 0.0001.

50 relapses in 35 AQP4-IgG+ patients and 26 relapses in 19 MOG-IgG+ patients were included. 12 ‘attack’ samples were collected within 30 days before symptoms onset (thus before attack treatment initiation) in AQP4-IgG+, and 3 in MOG-IgG+. 38% of AQP4-IgG+ relapses (19/50) and 38.5% MOG-IgG+ relapses (10/26) had at least a 2-fold increase (1 doubling dilution increase) in titres during relapse. The median titre change was a 1.25-fold increase (IQR 1–2) for AQP4-IgG+ and 1.5-fold increase (IQR 1–2) for MOG-IgG. If only those patients with a titre increase were considered, then they increased by a median of 2-fold (IQR 2–2.5) for AQP4-IgG+ and of 2-fold for MOG-IgG+ (IQR 1.5–2.4). Overall, at relapse, there was a significant increase in AQP4-IgG+ titres (*P*  *=* 0.0104) (Fig. 3A; Supplementary Fig. 1A) and MOG-IgG+ titres (*P*  *=* 0.012) (Fig. 3E; Supplementary Fig. 1E). Attack titres were higher compared to pre-attack (median time 7 months, IQR 4–10, for AQP4-IgG+, and 6 months, IQR 3–9, for MOG-IgG+) by a mean change of 0.49 in logarithmic scale in AQP4-IgG+ (coefficient β1​=0.49, Standard Error, SE = 0.18) and 0.53 in MOG-IgG+ (β1​=0.53, SE = 0.20). Lower titres at ‘pre-attack’ tend to have a larger increase during relapse in both cohorts (AQP4-IgG+ coefficient of Correlation, Corr, −0.244, MOG-IgG+ Corr −0.33). The titres increase was not influenced by relapse phenotype, the sequence of the attack (number of attack) (Supplementary Fig. 2A,B), attack EDSS at nadir, long-term IST during relapse and time of sample collection according to symptoms onset (before or after 30 days).

### Antibody titres decrease in serum from ‘attack’ to ‘post-attack’

105 clinical attacks (71 relapses, 3 onset attacks in relapsing patients and 31 onset attacks in monophasic patients) in 85 AQP4-IgG+ patients and 112 clinical attacks (36 relapses, 7 onset attacks in relapsing patients and 69 onset attacks in monophasic patients) in 105 MOG-IgG+ patients were included. 14 ‘attack’ samples were collected within 30 days before symptoms onset (thus before attack treatment initiation) in AQP4-IgG+, 4 in MOG-IgG+ . There was a significant decrease in titres in ‘post-attack’ samples (median time from attack 8 months, IQR 6–11 months) for both AQP4-IgG+ (*P*  *<*  *0.0001*) (Fig. 3B; Supplementary Fig. 1B) and MOG-IgG+ (*P*  *<*  *0.0001*) (Fig. 3F; Supplementary Fig. 1F). ‘Post-attack’ samples were lower compared to ‘attack’ samples by a mean change of 2.02 in logarithmic scale in AQP4-IgG+ (β1: −2.02, SE = 0.29) and 2.99 in MOG-IgG+ (β1=−2.99, SE = 0.31). Median titre change was a 4-fold decrease (two doubling dilutions; IQR 2–8-fold) for AQP4-IgG+ and a 4-fold decrease (IQR 2–13) for MOG-IgG+ . 76.2% AQP4-IgG+ attacks (80/105) and 78.6% MOG-IgG+ attacks (88/112) had more than 2-fold (1 doubling dilution) decrease in titres in ‘post-attack’ sample. Higher titres at clinical attack tend to have smaller decrease in both cohorts (Corr −0.228 for AQP4-IgG+, Corr −0.088 for MOG-IgG+).

AQP4-IgG+ clinical attacks with BS had lower attack titres compared to myelitis (*P*  *=* 0.002).

**Figure 4:**
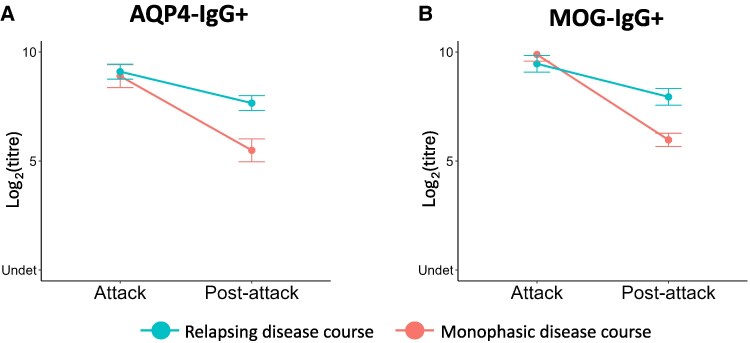
**Estimated marginal means of antibody titres change at post-attack according to disease course**. Estimated marginal means (EMM) of antibody titres (in logarithmic scale) at ‘Attack’ and ‘Post-attack’ in patients with a relapsing and a monophasic disease course, both in AQP4-IgG+ (*N* = 105 attacks, A) and MOG-IgG+ (*N* = 112 attacks, B) cohorts, showing higher ‘Post-attack’ titres in relapsing patients through *post hoc* analysis. The points represent the mean titres values at each time point. Error bars indicate the standard error of the means.

There was no difference in attack titres in patients with relapsing disease compared to those with monophasic disease. Both AQP4-IgG+ and MOG-IgG+ patients with a relapsing disease course had higher ‘post-attack’ titres compared to those with monophasic disease (AQP4-IgG+ *P*  *=* 0.0008, MOG-IgG+ *P*  *<* 0.0001) (Fig. 4A and B), likewise the titre reduction in post-attack sample was smaller in relapsing patients (*P*  *<* 0.0001 for both AQP4-IgG+ and MOG-IgG+).

In ‘post-attack’ sample, 29/112 (26%) MOG-IgG+ attacks—including 27 onset attacks and 2 relapses—had a decrease ≥6-fold (3 doubling dilutions) in titres. Among these, 25 attacks (86%) occurred in monophasic patients. Of the 83 attacks without a subsequent decrease of ≥6-fold 44 (53%) had a monophasic course. Monophasic MOG-IgG+ patients had an odds ratio (OR) of 11.1 (confidence of interval, CI, 95% 2.46–49.82) of having a decrease in titres ≥6-fold compared to relapsing ones. Similarly, 31/105 (30%) AQP4-IgG+ attacks had a decrease ≥6-fold (three doubling dilutions) in titres, with 19 (61%) of these attacks in monophasic patients. Of the 74 attacks without a subsequent decrease of ≥6-fold 12 (16%) had a monophasic course. Of the 31 monophasic AQP4-IgG+ patients: 19 had a ≥ 6-fold (three doubling dilutions) decrease, 9 an intermediate titre decrease (≥2-fold and <6-fold) and 3 had stable titres. Monophasic AQP4-IgG+ patients had an OR of 8.18 (CI, 95% 3.16–21.17) of having a decrease in titres ≥6-fold compared to relapsing ones.

In AQP4-IgG+ NMOSD age at onset and age at attack had a significant influence on antibody titre change (*P* < 0.0001, and *P* < 0.001, respectively), the higher the age the greater the titres reduction. This effect was not observed in MOG-IgG+ cohort. Attack EDSS and time since last attack did not have any effect on antibody titres in both cohorts. Time of sample collection according to symptoms onset (before or after 30 days) did not change titre reduction in ‘post-attack’ samples in both cohorts.

### Antibody titres are stable from year 2 to year 4

Fifty-four ‘remission 1’-‘remission 2’ sequences in 52 AQP4-IgG+ patients and 29 ‘remission 1’-‘remission 2’ sequences in 29 MOG-IgG+ patients were included. There was no significant change in titres in both cohorts (AQP4-IgG+ *P* = 0.142, Fig. 3C and Supplementary Fig. 1C; MOG-IgG+ *P* = 0.779, Fig. 3G and Supplementary Fig. 1G).

Forty ‘remission 2’-‘remission 3’ sequences in 39 AQP4-IgG+ patients and 15 ‘remission 2’-‘remission 3’ sequences in 15 MOG-IgG+ patients were included. There was no significant change in titres for both cohorts (AQP4-IgG+ *P* = 0.197, Fig. 3D and Supplementary Fig. 1D; MOG-IgG+ *P* = 0.344, Fig. 3H and Supplementary Fig. 1H).

### A ≥ 2-fold increase (one doubling dilution) in antibody titres is more frequent during relapse

**Figure 5:**
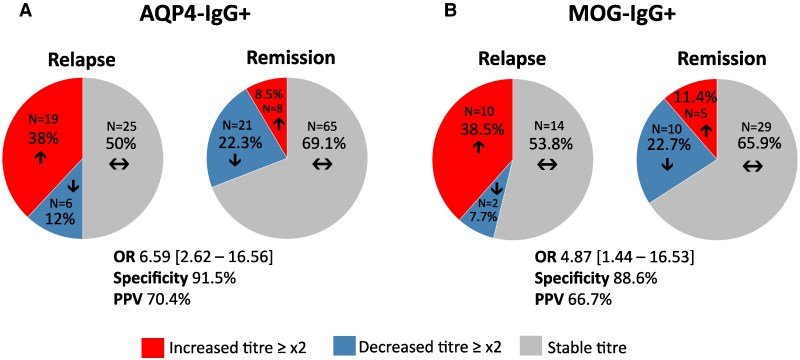
**Frequency of antibodies change during relapse and remission.** Frequency of antibodies change during relapse (sequence ‘pre-attack’—‘attack’) and remission (sequences ‘remission 1’—‘remission 2’ and ‘remission 2’—‘remission 3’) for AQP4-IgG+ (A) and MOG-IgG+ (B), focusing on increase of titres ≥ ×2, decrease ≥ ×2 and stable titres. Abbreviations: OR, odds ratio; PPV, positive predictive value.

In 67 AQP4-IgG+ patients, we identified 27 increases in titres in 144 pairs of serum samples. Of those, 19/50 from relapses (‘pre-attack’-‘attack’ sequences), 4/54 from ‘remission 1’-‘remission 2’ sequences and 4/40 from ‘remission 2’-‘remission 3’ sequences had a ≥ 2-fold increase in titres (1 doubling dilution; Fig. 5A). AQP4-IgG+ patients had an OR of 6.59 (CI, 95% 2.62–16.56) of having a ≥ 2-fold increase of titres during a relapse compared to a period of clinical stability. A ≥ 2-fold increase in titres had a 38% sensitivity, 91.5% specificity and a 70.4% positive predictive value (PPV) of being associated with a relapse. AQP4-IgG+ patients had an OR of 2.24 (CI 95% 1.11–4.54) of having unchanged titres during remission compared to relapse.

In 48 MOG-IgG+ patients, we identified 15 increases in titre in 70 pairs of serum samples. Of those, 10/26 were from relapses (‘pre-attack’-‘attack’ sequences), 4/29 from ‘remission 1’-‘remission 2’ sequences and 1/15 from ‘remission 2’-‘remission 3’ sequences had a ≥ 2-fold increase in titres (Fig. 5B). MOG-IgG+ patients had an OR of 4.87 (CI 95% 1.44–16.53) of having a ≥ 2-fold increase in titres during a relapse compared to a period of clinical stability. A ≥ 2-fold increase of titres had a 38.5% sensitivity, 88.6% specificity and a 66.7% PPV of being associated with a relapse. MOG-IgG+ patients had an OR of 1.66 (CI 95% 0.61–4.46) of having unchanged titres during remission compared to relapse.

### Long-term ISTs can reduce titres in AQP4-IgG+ and MOG-IgG+ patients during follow-up

**Figure 6:**
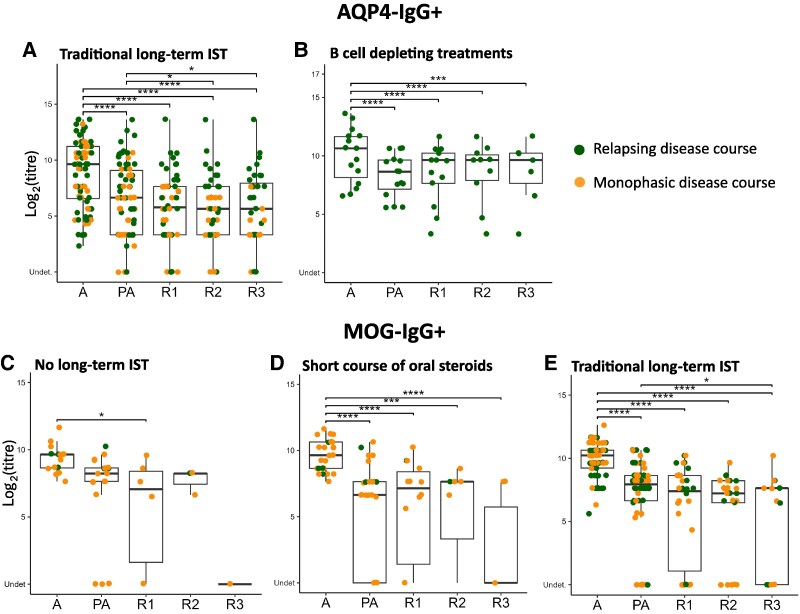
**Trend of antibody titres after clinical attack in AQP4-IgG+ and MOG-IgG+ cohorts divided by immunosuppressant treatment**. Trend of antibody titres after clinical attack in AQP4-IgG+ cohort (A, B) and MOG-IgG+ cohort (C–E), divided by immunosuppressant treatment (IST): no treatment (*N* = 18 attacks, C), short course of oral steroid (*N* = 28 attacks, D), traditional IST (*N* = 76 attacks, A; *N* = 64 attacks, E), and B-cells depleting treatment (*N* = 16 attacks, B). Each box plot shows statistical significance of antibody titres change by LMEM: ‘Attack’ versus ‘Post-Attack’ versus ‘Remission 1’ versus ‘Remission 2’ versus ‘Remission 3’. Each point represents the titre value (in log2 scale) of a single attack, coloured differently depending on the disease course (relapsing or monophasic). All significant time point comparisons are indicated with an asterisk, while comparisons not indicated are non-significant. Abbreviations: Undet, undetectable; LMEM, linear mixed effect model; A, attack; PA, post-attack; R1, remission 1; R2, remission 2; R3, remission 3. * = *P* ≤ 0.05, ** = *P* ≤ 0.01, *** = *P* ≤ 0.001, **** = *P* ≤ 0.0001.

Long-term ISTs were initiated, or dosage adjustments were made, following 92 AQP4-IgG+ clinical attacks: traditional long-term ISTs during 76 attacks, and B cell depleting agents during 16 attacks. A reduction in titres in the first year after the attack was observed in those who received long-term traditional ISTs and B cell depleting agents (Fig. 6A and B). When traditional long-term ISTs were being taken a further reduction in antibody titres was observed during follow-up (Fig. 6A). Dividing these attacks according to the disease course, only patients with monophasic course maintain a further reduction in antibody titres during follow-ups compared to relapsing patients (Supplementary Fig. 3A,B).

Long-term ISTs were initiated, or dosage adjustments were made following 92 MOG-IgG+ clinical attacks. A short course of oral steroids (<12 months, median time 7 months, IQR 4–9 months) were initiated/increased for 28 attacks (oral steroids were always kept at a dosage above 10 mg, excluding the short period of escalation or weaning). Traditional long-term ISTs were initiated/increased during 64 attacks. No MOG-IgG+ attacks received B cell depleting agents. 18 MOG-IgG+ attacks were treated only with acute treatment and did not receive any long-term ISTs. In those who received a short course of oral steroids (median time 7 months, IQR 4–9 months) there was a reduction in antibody titres in the first year after the attack (Fig. 6D). In those who received traditional long-term ISTs there was a reduction in antibody titres in the first year after the attack (Fig. 6E). In those who did not receive any long-term ISTs this reduction occurred later at year 2 (Fig. 6C).

### A higher proportion of serum MOG-IgG titres fall below the test cut-off

**Figure 7:**
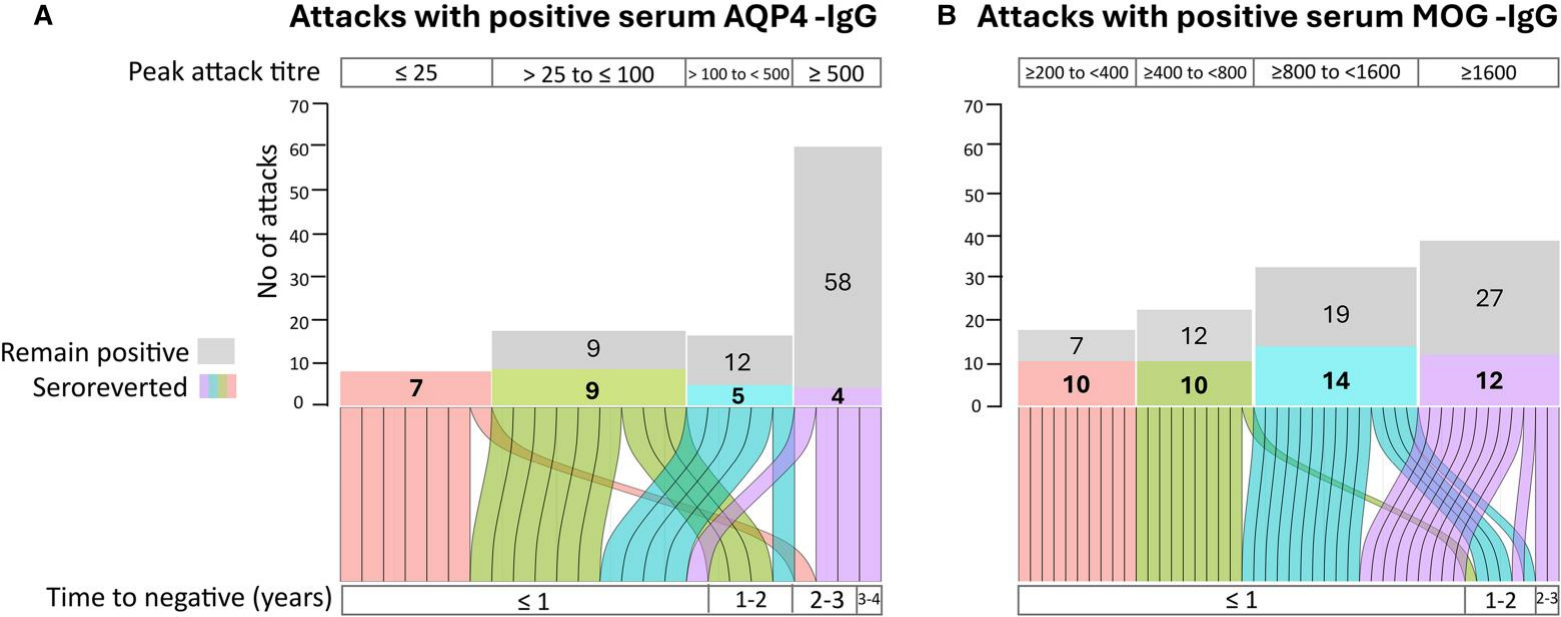
**Frequency of seroreversion below the test cut-off in AQP4-IgG+ and MOG-IgG+ cohorts**. On the top of the graph: numbers of attacks dropping to undetectable antibodies in at least one follow-up serum sample (post-attack, remission 1, remission 2 or remission 3), after an attack with positive antibodies, in AQP4-IgG+ (A, *N* = 25/92 patients seroreverted) and MOG-IgG+ (B, *N* = 46/111 patients seroreverted) cohorts, compared to all attacks occurred, divided according to the range of attack titres. On the below of the graph: alluvial plot of time to conversion to a negative serostatus according to the different value of titre during the attack. Time to seroreversion were classified as: ≤1 year (seroreversion at sample ‘post-attack’), ≤2 years (seroreversion at sample ‘remission 1’), ≤3 years (seroreversion at sample ‘remission 2’) and ≤4 years (seroreversion at sample ‘remission 3’).

25/92 AQP4-IgG+ patients became negative in at least one follow-up serum sample after a median time of 11 months (IQR 7–20 months) from positive clinical attacks: 11/25 (44%) patients had relapsing disease course (becoming negative after relapses) and 14/25 (56%) had a monophasic one (becoming negative after onset and unique attack). Of these 25 AQP4-IgG+ attacks, 2/25 (8%) were treated with chronic oral steroids, 3/25 (12%) with rituximab and all the other patients (20/25, 80%) with traditional ISTs. 5/121 AQP4-IgG+ relapses occurred with serum antibody titres undetectable in the attack sample (within 30 days of the attack and before an increase in treatment): in four cases, it was the last relapse reported in the patient (median number relapse 4.5, IQR 3.5–5, median time from onset 3 years, IQR 1.75–6.75 years), one patient had another relapse 5 years later. All five patients with negative serum test at relapses had samples at all time points, with a total time of follow-up from relapse of 48 months (IQR 39–48 months). Only 1/5 relapsing patient seroconverted to positive in a subsequent follow-up at sample ‘remission 2’ (26 months after the seronegative attack result, with a titre of 1:25, and seroreverted again to negative at sample ‘remission 3’). Another patient seroconverted to positive in a subsequent follow-up sample outside the time frame analysed in this study (after 8 months from sample ‘remission 3’, with a titre 1:20, and then back to negative in a subsequent sample). Most of the AQP4-IgG+ patients (64%) whose serum AQP4-IgG fell below the cut-off had low attack titres ≤ 100 (Fig. 7A). A lower proportion (7%) of patients with higher attack titres (≥500) became undetectable and this took longer: fewer patients (3) with median attack titres of 1900 (IQR 600–3200) fell below the test cut-off in a median time of 30.5 months (IQR 23.7–33.8) versus the median time of 10 months (IQR 5.5–16.0 months) for seroreversion in patients with low attack titres (median titre 25 (20–25)). One patient seroconverted to positive in a subsequent follow-up sample outside the time points of the time-frame considered in the study, after 84 months from the attack. 46/111 MOG-IgG+ patients became seronegative in at least one follow-up serum sample after a median time of 10 months (IQR 7–12.5 months) from a MOG-IgG+ positive serum test at the clinical attack (Fig. 7B): 37/46 monophasic patients (80.4%) and 9/46 relapsing patients (19.6%). 21/46 (46%) received a traditional IST (including 17 receiving oral steroids for more than 1 year), 18/46 (39%) received short course of oral steroids (<12 months) and 7/46 (15%) did not receive any long-term treatment. 4/37 monophasic patients and 1/9 relapsing patients seroconverted to positive in a follow-up sample in a median time of 14 months (IQR 14–15 months) after the negative result. None of these patients seroconverted to positive in subsequent follow-up samples outside the time frame of the study. Just 1/134 (0.7%) MOG-IgG+ relapses occurred with serum antibody titres below the cut-off in the sample attack. Most of the seroreverting patients (38/46, 83%) dropped to negative in the first year after the clinical attack, independently of the titre during the attack.

## Discussion

We analysed the dynamics of antibody titres across clinical phases in a large cohort of both AQP4-IgG+ NMOSD and MOG-IgG+ MOGAD patients. Antibody titres are highest in attack samples. Titres increase from pre-attack to attack samples in both disease groups in 38–39% of attacks, increasing by a median of 1.25 (AQP4-IgG+) or 1.5-fold (MOG-IgG+) and decrease by 2-fold afterwards. Increases in antibody titres were observed in 9–11% of remission phase samples meaning that in 66.7–70% of cases such increase is associated with a relapse. Reductions in antibody titres are more frequent post relapse compared to pre relapse which may relate to changes in immune therapies. These findings have important implications for understanding disease pathophysiology and informing clinical management. Serial antibody testing for predicting relapses in clinical practice can be a valuable but not decisive support.

These observations support the use of both serum antibodies as an adjunct in monitoring disease activity. Specifically, our findings highlight the potential utility of these antibodies as additional support in dealing with clinical complex scenario, like where patients have de-escalated their treatment, shown inconsistent adherence to prescribed therapies, or where clinical ambiguity raises concerns about whether a genuine relapse has occurred. However, the additional value of antibody titres in monitoring disease activity has to contend with several difficulties in clinical practice, such as the limited availability worldwide of a gold-standard test (live CBA) and access to concomitant paraclinical tests (such as MRI or ophthalmic evaluation) in the presence of increased titres. To better evaluate the role of antibodies during acute disease activity, future research is needed to assess the antibodies binding capacity (affinity and avidity) and of other IgG subclasses during relapses.

The observation that the proportion of patients with elevated antibodies levels during relapse and remission are so similar in both AQP4-IgG NMOSD and MOGAD cohorts is interesting. We might have predicted titres to be more aligned with relapses in AQP4-NMOSD relapses for two reasons: (i) because the assay is ‘cleaner’ with less overlap with control sera and (ii) the antibodies are accepted to be more directly pathogenic. In MOGAD, there may be multiple pathogenic mechanisms, some of which unrelated to the antibodies, hence our surprise the data were so similar. However, many MOGAD patients will not be on long-term immune suppressants and AQP4-NMOSD patients will be which may be a confounder.

We have shown how titres remain higher in those who have a relapsing disease course. The relevance of AQP4-IgGs titres in following disease activity has previously shown mixed results.^4,14^ Negative studies may be partly due to either limited numbers of samples, fixed CBA, or to lack of longitudinal assessments to allow individual patient results to be linked. Cross sectional studies on paired samples using single tests have demonstrated a reduction in titres during remission,^15^ hinting at variation in titre with clinical course within individuals. Previous negative studies on changes in titres between relapse and remission are likely due to differences in study design, test system used, most often using unpaired samples with limited total sample numbers. In our study using paired samples and a single assay that can at best detect a 50% difference in serum antibody levels between samples, we identified 40% of relapses in both AQP4-IgG NMOSD and MOGAD. This suggests that a test that can accurately measure smaller differences in serum titres may be even more useful. Leading on from this, the meta-analysis^16^ not showing an effect is not surprising as it combined data from studies which included different techniques such as ELISA, CBA, FIPA and flow cytometry, each with different units, sensitivities and specificities. Given the well-known pathogenic role of AQP4-IgGs produced peripherally, the increase of the antibody-producing plasmablasts and plasmacells signatures during attacks, as shown in N-MOMENTUM trial,^17^ could reinforce the idea of the role of antibodies during attacks and the need of improve the way of detecting them. Moreover, in this study patients with higher AQP4-IgGs titres at day 1 had increased annualized relapse rate. Majed *et al.*^4^ and Jitprapaikulsan *et al*.^7^ were unable to establish a predictive role for AQP4-IgGs in the disease course, likely due to the limitations of the 10-fold dilution approach used in their titrations, which may have overlooked smaller titre changes. Using one in two dilutions as in our centre is likely to be more sensitive to smaller titre changes. Perhaps a fully quantitative method will prove even more useful. The higher frequency of seroreversion in AQP4-IgG+ patients compared to Majed *et al*.^4^ (11% versus 29%) may be attributed to the slightly shorter observation period in Majed study (median interval of 1.7 years, IQR 0.5–3.7, versus 2 years, IQR 1–3). Here, we show a greater reduction in antibody titres during the first year of remission in AQP4-IgG+ patients with a monophasic disease course compared to patients with a relapsing course despite similar therapies. NMOSD-AQP4 + patients who maintain high antibody titres after onset have a higher probability of relapse (*P*  *=* 0.0008), so AQP4-IgGs titres during remission could guide treatment choice and target patients who can be treated with more aggressive ISTs early or who cannot be weaned from treatment.

Monophasic MOGAD patients tend to have lower titres during remission and to drop below the assay cut-off within 1 year from onset. A 6-fold reduction in titres was observed predominantly in monophasic patients (OR 11.1), aligning with previous literature.^18^

Thus, serum AQP4-IgGs and MOG-IgGs titres could serve as indicators of an increased risk of relapse in a proportion of individual patients. Several studies have already shown how titres during attacks are not correlated with clinical outcomes and residual disability both in MOGAD and NMOSD,^19,20^ but our study highlights utility of remission titres to suggest individuals more likely to relapse and so guide for clinical management.

We suggest at least a 2-fold increase (a single doubling dilution) in serum antibody titres may be a useful guide for relapse risk, supported by an OR of 6.59–4.87. We noted a 2-fold increase in titres raises the risk of relapse although not all patients whose titres increase have a re-exacerbation of the disease. On the other hand, no titre increase was less discriminatory in distinguishing those who relapse from those who were stable (OR 2.24–1.66). The higher frequency of a 2-fold increase or more in antibody titre during a relapse (38–38.5% versus 8.5–11–4%) justifies the use of these biomarkers for aiding in the management decisions and particularly in confirming active disease. The high specificity (91.5–88.6%) and moderate PPV suggest that a 2-fold increase can indicate a relapse, making it a helpful but not definitive marker. Not every increase in serum titres, for both AQP4-IgG+ and MOG-IgG+, is accompanied by a clinical relapse, as other factors may play a role.^21^ Future longitudinal studies are needed to determine whether these asymptomatic increases in serum titres, when not associated with treatment modulation, are associated with subclinical disease activity, such as new MRI lesions or increases in biomarkers of neurodegeneration, like neurofilament light chain (Nfl) or glial fibrillary acid protein (GFAP).

We observed a clear difference in the dynamics of serum antibody titres in AQP4-IgG+ NMOSD and MOG-IgG+ MOGAD from a positive attack to a status of undetectable antibodies. The AQP4-IgG levels in the serum of 25/92 AQP4-IgG+ patients on treatment fell below the cut-off after 11 months. When the attack titre was low (≤25), 100% fell below the test cut-off after 10 months. Patients with high attack titres (≥500) fell below the test cut-off less frequently (7%) and took longer to serorevert (with a median time of 30.5 months). So, in AQP4-IgG+ serum seroreversion is associated with attack titres. All seroreverted patients were treated with ISTs, previous literature suggests that this seroreversion is not sufficient to wean-off treatment.^22^

Serum MOG-IgG levels in 46/111 MOG-IgG+ patients fell below the test cut-off after a median of 10 months. Of these 83% (38/46) seroreverted below the cut-off within 1 year after the attack, 32/38 (84%) of whom had a monophasic disease course, supporting previously published data that patients who serorevert are less likely to relapse. Of the 46 patients, 35 (76%) were treated with steroids (with a different time of dose reduction) support the role of a prolonged course of steroids in giving a better prognosis in MOGAD.^23^ Additional studies investigating the disease course and the natural history of MOGAD are needed to understand other possible mechanisms driving its relapsing course. The different dynamics of AQP4-IgGs and MOG-IgGs after positive serum attacks are summarized in Supplementary Table 2.

There are limitations and strengths of our study. First, its retrospective nature presents challenges due to the varying time points of sample collections. These observations are likely only relevant where live CBA are used with 2-fold dilutions and further studies are needed using the fixed CBA with similar dilutions. The limited availability of samples and the absence of samples at all time points may have caused us to miss important changes. To truly understand antibodies dynamics throughout the disease course, longitudinal prospective studies with fixed collection times are needed. Additionally, there is a limited numbers of samples for the ‘pre-attack’-‘attack’ sequence compared to the relatively higher number of samples during remissions. This imbalance could underestimate the PPV value and OR of observing a ≥ 2-fold increase during a relapse. The low number of AQP4-IgG+ patients treated with B cell depleting agents may be responsible for not showing a significant effect with these types of treatment. Finally, we did not include acute attack treatments (such as intravenous methylprednisolone or plasma exchange) in our analysis; however, we assumed the effect on antibody levels was most likely to have worn-off after 3 months. Moreover, no changes were observed considering the time of ‘attack’ sample collection (before or after 30 days symptoms onset, and so start of acute treatment), suggesting short acute treatment does not influence this trend.

Our test system is semi-quantitative meaning on average we only see a change in titre when there is an increase or decrease of 50% of the total target specific antibody levels. More subtle but consistent changes are not captured. Additionally, a change of one dilution in low titre samples involves fewer antibody molecules than a similar change in a high titre sample. So identical changes in antibody amounts will give different results depending on their context.

One of the strengths is the unique comparison of the dynamics of both MOG-IgGs and AQP4-IgGs titres during different phases of disease course, highlighting the differences between the immune mechanisms in the two diseases. Additionally, the inclusion of a large cohort of patients and numerous clinical attacks enhances the robustness of our findings. The availability of extensive serum samples collected at multiple time points further reinforces the study's significance. The use of the gold-standard technique to assess antibody titres (live CBA) is a strength of our study; techniques such as fixed CBA are more common in clinical practice, but much less sensitive, for example a recent publication from the Department of Neurology at Johns Hopkins University suggests a commercial fixed test has a sensitivity of 72% for AQP4 and 46% for MOG when compared to a live test run at the Mayo Clinic which had a sensitivity of 95% for MOG-IgG1 and 97% for AQP4, so their use is much less accurate for monitoring patients.^24^ Maintaining consistency in antibody titres is difficult with so many variables in repeat testing. The data for this paper was generated visually by microscopy, captured by one scientist (M.W.) using the same in-house method and evaluated on the same microscope over 16 years.

## Conclusion

In 63–61% of longitudinal serum samples from patients with AQP4-IgG or MOG-IgG, there is no increase in antibody titre independently of disease phases. When there is an increase, it is most often at relapse: 38–39% of relapse samples and 9–11% of remission samples show at least a 2-fold increase. Hence, an increase in titres should alert the clinician to a 5–6 times increased risk of having a relapse, but in the majority of cases a relapse is not associated with a serum titre increase as evaluated by titres on a live CBA. Serum antibodies titres can have an important role as adjunct in clinical management, especially in front of a pseudo-relapse. In both MOGAD and NMOSD-AQP4-IgG+ disease, those with lower remission titres during the first year after the attack tend to have a monophasic disease, suggesting titres during remission may be useful markers for treatment choice and clinical management. Seroreversion to negative in MOGAD patients is more frequent in monophasic patients, independent of attack titre, while seroreversion in NMOSD-AQP4 + patients is attack titre dependent. Our study supports the utility of serological surveillance to inform clinical management, especially in the first year after the initial attack and when a relapse is suspected. Longitudinal prospective studies with quantitative antibody measurement will better evaluate the utility of antibody titres.

## Supplementary Material

fcaf312_Supplementary_Data

## Data Availability

The data that support the findings of this study are available from the corresponding author, upon reasonable request. The R code used for statistical analysis and figure generation is available in the Supplementary material.
