# Supplementary material for: Quantitative analysis of aquaporin-4 (AQP4) and myelin oligodendrocyte glycoprotein (MOG) antibodies titres: correlation with relapses
Source: Brain Commun. 2025 Aug 22;7(5):fcaf312. doi: 10.1093/braincomms/fcaf312 (PMC12418092; doi:10.1093/braincomms/fcaf312)
Supplement: fcaf312_Supplementary_Data [file fcaf312_supplementary_data.zip › Supplementary_material.docx]

**Supplementary figures**


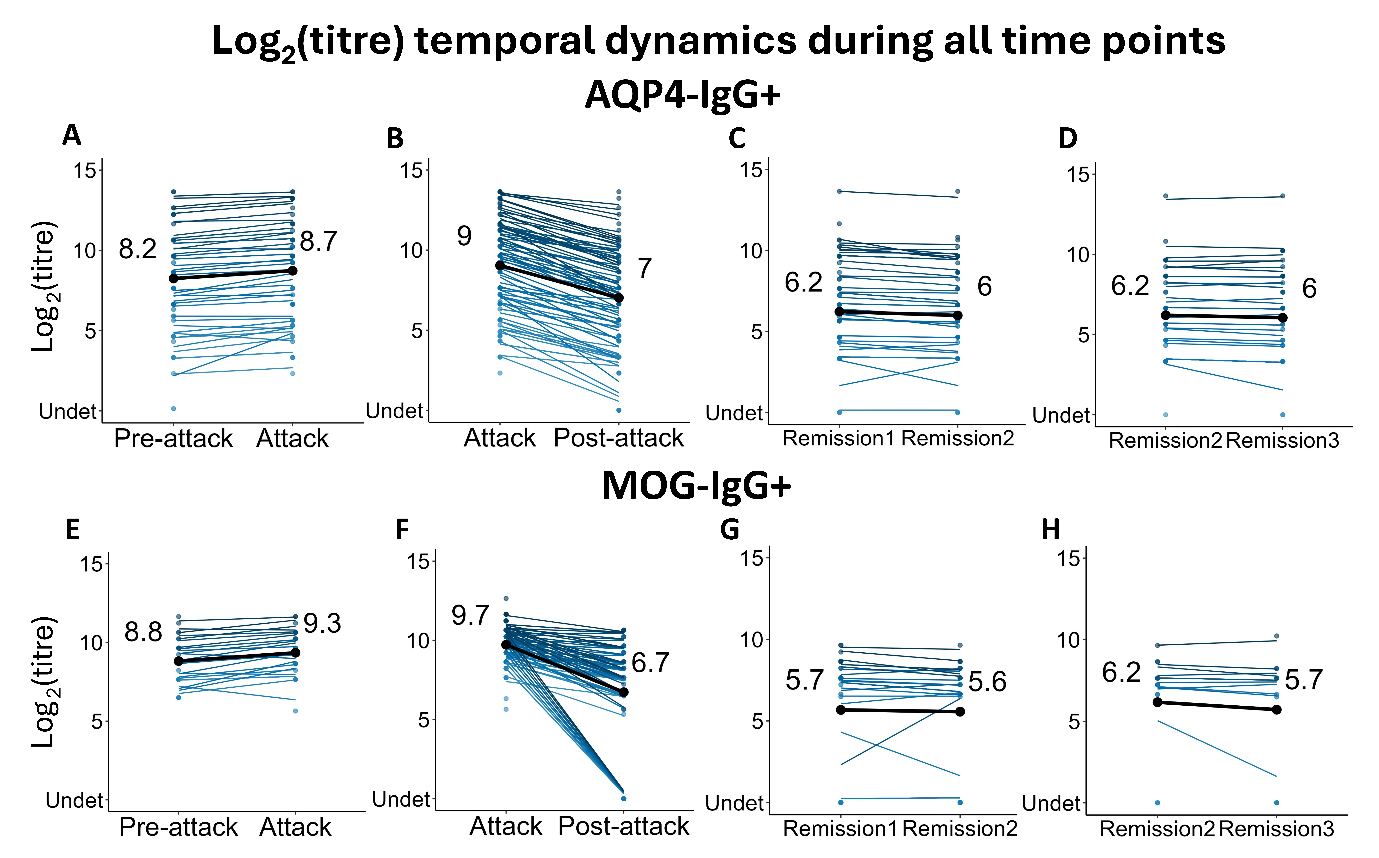


- e**Figure 1:** **Trend of antibody titres change during attacks and clinical stability**. Trend of antibody titres change during attacks and clinical stability for both AQP4-IgG+ and MOG-IgG+: ‘Pre-attack’ vs ‘Attack’ (N=50 relapses for AQP4-IgG+,A, N=26 relapses for MOG-IgG+,E), ‘Attack’ vs ‘Post-attack’ (N=105 attacks for AQP4-IgG+,B, N=112 attacks for MOG-IgG+,F), ‘Remission 1’ vs ‘Remission 2’ (N=54 sequences for AQP4-IgG+,C, N=29 sequences for MOG-IgG+,G), ‘Remission 2’ vs ‘Remission 3’ (N=40 sequences for AQP4-IgG+,D, N=15 sequences for MOG-IgG+,H). Points represent the observed titres (in log2 scale) for each attack across different time points. Lines represent the fitted titre values predicted by the LMEL for each attack (the model's prediction based on the fixed and random effects). Each unique colour represents a different attack. Black points represent titres mean value, indicated on each side. Abbreviations: Undet: undetectable; LMEL: Linear Mixed Effect Model.


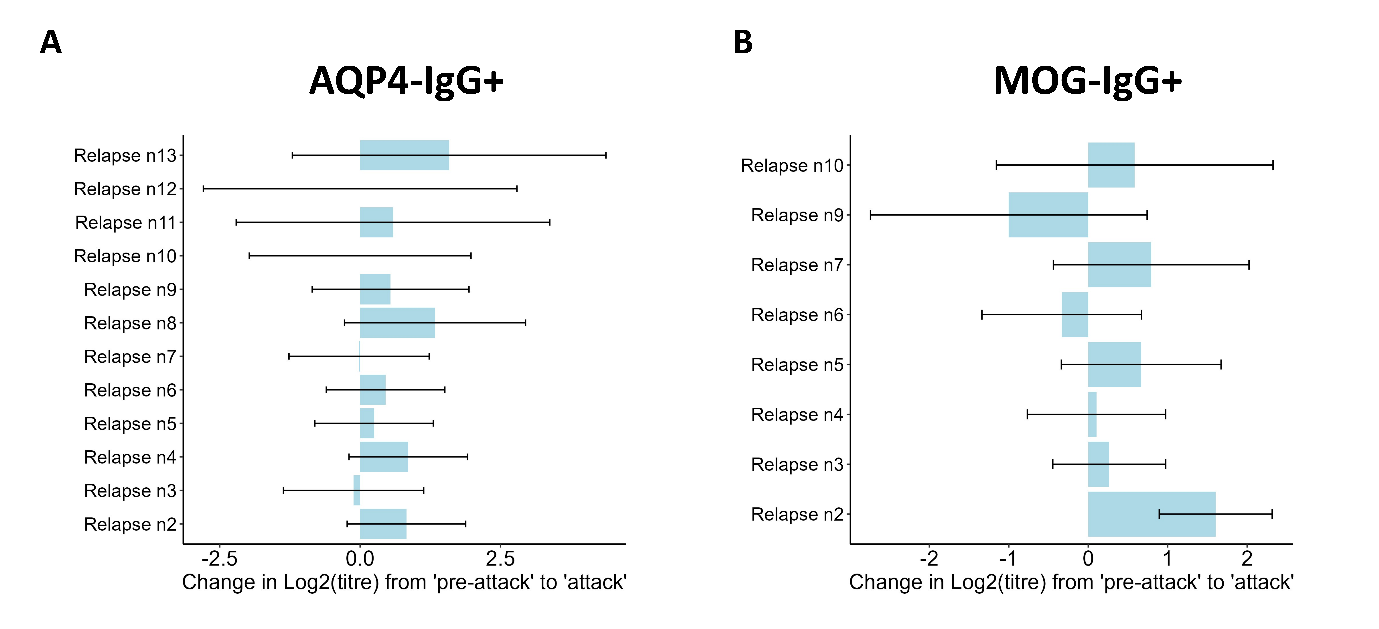


- **eFigure 2**: **Bar plot of the estimated change of titres during attacks according to number of relapse**. Bar plot of the estimated change of AQP4-IgGs (N=105 attacks, A) and MOG-IgGs (N=112 attacks, B) titres (in logarithmic scale) from ‘Pre-attack’ to ‘Attack’ according to different number of relapse through post-hoc analysis. Each horizontal bar indicate the magnitude of the estimated change of titres. The error bars extending from each bar represent the 95% confidence intervals of the estimates.


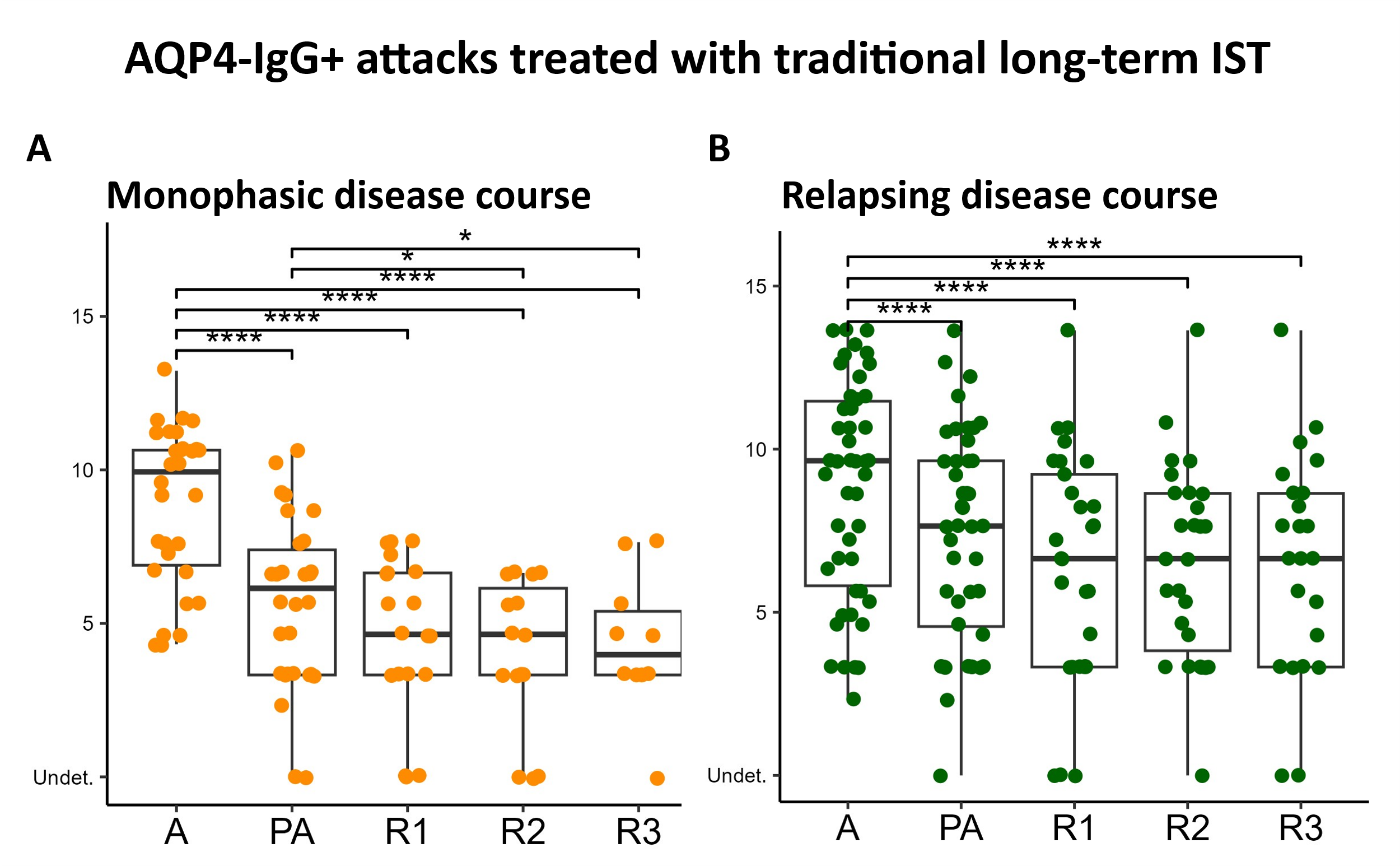


- e**Figure 3**: **Trend of antibody titres after clinical attack in AQP4-IgG+ cohort according to immunosuppressant treatment and disease course**. Trend of antibody titres after clinical attack in AQP4-IgG+ cohort treated with traditional long-term immunosuppressant treatment (IST), divided by disease course in monophasic (N=30 attacks, A) and relapsing (N=46 attacks, B) patients. Each box plot show statistical significance of antibody titres change by LMEM: ‘Attack’ vs ‘Post-Attack’ vs ‘Remission 1’ vs ‘Remission 2’ vs ‘Remission 3’. Each point represents the titre value (in log2 scale) of a single attack. All significant time point comparisons are indicated with an asterisk, while comparisons not indicated are non-significant. Abbreviations: Undet: undetectable; LMEM: Linear Mixed Effect Model; A: Attack; PA: Post-attack; R1: Remission 1; R2: Remission 2; R3: Remission 3. Legend: * = p≤0.05, ** = p ≤0.01, *** = p ≤0.001, **** = p ≤0.0001.

**Suppl. Table 1. Characteristics of serum samples**

|  | **AQP4-IgG+** | | | **MOG-IgG+** | | |
| --- | --- | --- | --- | --- | --- | --- |
| **Samples** | **N** | **TFA, median (IQR)** | **Titre, median (IQR)** | **N** | **TFA, median (IQR)** | **Titre, median (IQR)** |
| Pre-attack | 50 | 7 months (4-10) | 400 (65-1600) | 26 | 6 months (3-9) | 400 (200-800) |
| Attack | 117 | 14 days (9-21) | 800 (100-2400) | 127 | 17 days (11-23) | 800 (400-1600) |
| Post-attack | 105 | 8 months (6-11) | 200 (25-800) | 112 | 8 months (6-11) | 200 (100-400) |
| Remission 1 | 70 | 19 months (16-23) | 100 (10-400) | 50 | 20 months (16-22) | 170 (0-400) |
| Remission 2 | 57 | 32 months (28-35) | 100 (10-400) | 36 | 32 months (28-34) | 200 (100-300) |
| Remission 3 | 48 | 43 months (40-48) | 100 (10-400) | 21 | 44 months (40-47) | 100 (0-200) |

Abbreviations: AQP4 = acquaporin-4, MOG = myelin oligodendrocyte glycoprotein, IgG = immunoglobulin G, TFA = time from attack, IQR = interquartile range.

**Suppl. Table 2. AQP4-IgGs and MOG-IgGs dynamics after serum positive attacks**

|  | **AQP4-IgG+** | **MOG-IgG+** |
| --- | --- | --- |
| **Titre to seronegative status** | More likely at low titres (≤100) | Initial titre not predictive |
| **Time to seronegative status** | Higher titres take longer to seroconvert | Most seroconvert in 1 year |
| **Frequency of seronegative status during disease course** | Usually related to treatment | Usually part of the natural history of the disease |
| **Impact of seronegative status in disease course** | Not useful to predict relapses off treatment [1] | Less likely to relapse [2] |

Abbreviations: AQP4 = acquaporin-4, MOG = myelin oligodendrocyte glycoprotein, IgG = immunoglobulin G. References: [1] Kessler RA, Mealy MA, Jimenez-Arango JA, Quan C, Paul F, López R, Hopkins S, Levy M. Anti-aquaporin-4 titer is not predictive of disease course in neuromyelitis optica spectrum disorder: A multicenter cohort study. Mult Scler Relat Disord. 2017 Oct;17:198-201. doi: 10.1016/j.msard.2017.08.005. Epub 2017 Aug 16. PMID: 29055457.\n. [2] Wendel EM, Thonke HS, Bertolini A, et al (2022) Temporal Dynamics of MOG Antibodies in Children With Acquired Demyelinating Syndrome. Neurol Neuroimmunol Neuroinflamm 9:"https://doi.org/10.1212/NXI.0000000000200035.
